# Pyrethroid resistance in the dengue vector *Aedes aegypti* in Southeast Asia: present situation and prospects for management

**DOI:** 10.1186/s13071-018-2899-0

**Published:** 2018-06-04

**Authors:** Zheng Hua Amelia-Yap, Chee Dhang Chen, Mohd Sofian-Azirun, Van Lun Low

**Affiliations:** 1 Tropical Infectious Diseases Research & Education Centre (TIDREC), University of Malaya, 50603 Kuala Lumpur, Malaysia; 2 Institute of Biological Sciences, Faculty of Science, University of Malaya, 50603 Kuala Lumpur, Malaysia

**Keywords:** Pyrethroids, Insecticide resistance, Knockdown resistance, *kdr*, Metabolic-mediated resistance

## Abstract

Human arboviral diseases transmitted by *Aedes aegypti* such as dengue, Zika, chikungunya and yellow fever remain global public health threats to date. Of these diseases, dengue fever is particularly prevalent in Southeast Asia. Relentless vector control efforts are performed to curtail disease transmissions through which pyrethroid insecticides are broadly used as the first line of defense to control *Ae. aegypti*, especially in the course of disease outbreaks. Here, we compile the largest contemporary database for susceptibility profiles and underlying mechanisms involved in *Ae. aegypti* resistant to pyrethroids in Southeast Asia. The extensive use of pyrethroids inevitably elicit different levels of resistance to numerous populations despite the presence of geographical isolation. The most common mechanisms of pyrethroid resistance that have been identified in *Ae. aegypti* includes mutations in the voltage sensitive sodium channel gene (*Vssc* gene) and metabolic-mediated insecticide resistance. *Aedes aegypti* develops resistance to pyrethroids by acquisition of one or several amino acid substitution(s) in this *Vssc* gene. Enzymes involved in metabolic-mediated detoxification (i.e. monooxygenases, glutathione-S-transferases and esterases) have been reported to be related to pyrethroid resistance but many specific contributory enzymes are not completely studied. An inadequate amount of data from some countries indicates an urgent need for further study to fill the knowledge gaps. Perspectives and future research needs are also discussed.

## Background

Dengue virus (DENV) and other arboviruses such as Zika virus, chikungunya virus and yellow fever virus are transmitted by *Aedes aegypti*. This highly adapted species thrives in urban and suburban areas. Effective control of *Ae. aegypti* is absolutely challenging when this species has deep-rooted and spread extensively in disease-endemic regions. With a lack of safe and effective treatment or vaccine for *Ae. aegypti*-borne diseases to date, tactics in suppressing *Ae. aegypti* continue to be the cornerstone in preventing dengue transmission and outbreak control.

An effective vector prevention to transiently remove *Ae. aegypti* involves several strategies such as eliminating potential mosquito breeding sites, applying chemical insecticides and biological control. However, the most extensively practiced control for *Ae. aegypti*, owing to its high efficacy in regulating the populations with relatively rapid action, is the application of chemical insecticides. The use of multiple classes of synthetic insecticides has been largely practiced in vector control strategies, with pyrethroid-based formulations massively dominating the insecticide market worldwide.

To the authors’ knowledge, there is no study to address the overall prevalence of pyrethroid resistance in *Ae. aegypti* in Southeast Asian countries. Therefore, this systematic review was carried out to determine related present situation to deliver datasets which will require in vector control management on improving public health of human population.

## Burden of pyrethroid resistance in *Aedes aegypti*

Geographical expansion of *Aedes aegypti* has fostered the incidence of dengue fevers (DF) to gain an apparent increase worldwide, which put an estimation of almost half of the world’s population at stake for the disease [1]. The most extensively practiced control for *Ae. aegypti*, owing to its high efficacy in regulating the populations with relatively rapid action, is the application of chemical insecticides [2]. Pyrethroid insecticides are currently being used extensively to control *Ae. aegypti* adults worldwide and constitute approximately 17% of the worldwide insecticide market share and 1.4 USD billion of global trades [3]. In general, pyrethroids are neurotoxins which modify the normal function of insect nerves and cause disruption at the *Vssc* gene by depolarizing neurons [4, 5], paralyzing and eventually killing the insect. Deltamethrin, cypermethrin, cyfluthrin, lambda-cyhalothrin, permethrin, alpha-cypermethrin, pyrethrum, bifenthrin, d-phenothrin, z-cypermethrin and etofenprox are the major types of pyrethroids used, and that their treatments usually involve either residual or space spray [6]. Narrowing down to Southeast Asia, deltamethrin, permethrin, alpha-cypermethrin, cyfluthrin and lambda-cyhalothrin are commonly employed in suppressing *Ae. aegypti*, particularly in Malaysia [7–9], Indonesia [10–12], Singapore [13–15] and Thailand [16–18]. All the on-shelf insecticides available for vector control are obliged to apply cautiously to delay worldwide evolution of mosquito resistance.

Currently, insecticide resistance is one of the most severe issues facing mosquito control agencies. The loss of efficacy of pyrethroids may consequently bring high possibility of operational failure of *Ae. aegypti*-borne diseases control and subsequently lead to increased disease transmission. This will cause even larger capital to be invested into mosquito control programmes by the government in return, for examples, the development of new insecticides in terms of labors and costs, which may cause direct socio-economic impact to a country. Other than the commonly-reported adverse impacts on public health and environment, reports particularly on the economic cost of pyrethroid resistance in *Ae. aegypti* in Southeast Asia have not been presented. This is in large part due to the progression of operating and resource costs throughout the vector control programme since the first occurrence of control problems until complete control failures have not been described. To expedite the progress and execution of scientifically-sound policies of insecticide use, economic costs in Southeast Asian context are vital to be studied.

A need of new paradigm or choice to control *Ae. aegypti* is undeniably urgent, provided that the current vector control strategies in Southeast Asia resulted in the discovery of insecticide-resistant mosquito populations. The ultimate goal of a vector control programme should focus on the establishment of an efficient government-community partnerships that the community plays a fundamental character in implementing these programmes. However, it should also be stressed that the success of these programmes can only be accomplished if both parties are committed to implement procedures in assisting community participation in *Ae. aegypti* control.

## Pyrethroid resistance mechanisms

Physiological resistance and behavioral avoidance of *Ae. aegypti* to insecticides are two integral responses ascribed to the intensified implementations of chemical-based control programmes. Physiological resistance implies the survival of mosquito victims under the exposure to chemical insecticides which would normally lead to complete death, comprising the most well-documented mechanisms conferring resistance against pyrethroid insecticides in *Ae. aegypti* such as insensitivity of target site which causes knockdown resistance (*kdr*) [19]; and metabolic detoxification *via* mixed function oxidases (P450 mediated monooxygenases), esterases and glutathione S-transferases (GSTs) [20]. Behavioral avoidance is the development of any change in the behavior of a mosquito to decrease the exposure of toxic substances or to escape lethal effect of insecticide, but this mechanism is easily overlooked [20]. Other than the aforementioned resistance mechanisms, insecticide resistance in *Ae. aegypti* is also mediated by cuticular resistance. Notes on each mechanism are described as follows.

### Behavioral avoidance

In-depth behavioral avoidance studies in *Ae. aegypti* are scarce across Southeast Asia until now. A change in mosquitoes’ feeding or resting behavior to minimize exposure to insecticides, this mechanism is generally categorized into direct contact excitation and non-contact spatial repellency [20]. Direct contact excitation (irritancy) is a phenomenon when mosquitoes escape from insecticide-exposed environment upon physical contact, while non-contact spatial repellency is when mosquitoes leaving the toxic area even before getting contact with a treated surface. Related behavioral avoidance studies of *Ae. aegypti* exposed to a range of pyrethroids have been inspected in Thailand, projected that average to strong irritancy have demonstrated in all tested populations when compared to repellency [21–24]. Despite the importance of behavioral responses of mosquitoes to insecticides contribute to the efficacy of chemicals, the relative complications of the assay designs, for instance, problems in differentiating between contact excitation and non-contact spatial repellency responses exerted in mosquitoes, lacking ideal statistical methods in analyzing data, and difficulties in introducing and removing test specimens, cause a dearth of information in this mechanism.

### Cuticular resistance

Cuticle thickening is implicated in insecticide resistance by reducing the uptake of the insecticide that reaches the target site in response to the modification of chemical composition of the cuticle. In mosquitoes, cuticular resistance is often pointed out but related studies are insufficient. A recent study revealed that this mechanism may play a major role in the development of resistance where it normally happens simultaneously with other mechanism(s) [25], causing resistance to single or multiple insecticides [26]. It has been reviewed elsewhere that cuticle thickening is associated with metabolic detoxification whereby thicker cuticle causes gradual insecticide absorption rate that will increase the effectiveness of metabolic detoxification in *Anopheles funestus* [27]. Moreover, it is crucial to take note that insects with cuticular resistance will display resistance level of not more than 3-fold in comparison to susceptible insects, but the co-occurrence of other resistance mechanism will lead to a surge in insecticide resistance level markedly [28]. This is demonstrated by *Anopheles gambiae* in Benin [29] through which overexpression of cuticular genes and P450 genes gave rise to a relatively high resistance level. Cuticular resistance to pyrethroids is also characterized in *Ae. aegypti* [30] but there is a lack of similar report documented in Southeast Asia. The fact that this least-understood mechanism may play substantial role in resistance urges for immediate attention in investigating, particularly in pyrethroid-resistant *Ae. aegypti*.

### Knockdown resistance (*kdr*)

Target site resistance in mosquitoes is related to either single or multiple mutations in target genes; for example, the *Vssc* gene which leads to *kdr*, mutations in the acetylcholinesterase (*Ace-1*) gene and GABA receptors [31, 32]. The most well-studied target site resistance for *Ae. aegypti* is *kdr* because it confers resistance against pyrethroids. The *Vssc* gene comprises four homologous domains and each of them contains six hydrophobic subunits (segments 1–6). In the past few decades, numerous mutations in the *Vssc* gene have been documented to be linked to *kdr* in many insect disease vectors. The single point L1014F mutation in the domain II segment 6 (IIS6) was the foremost mutation associated with pyrethroid resistance identified. Thereafter, different substitutions of C, F, H, S or W at this position have been reported in a few mosquito species in the genera of *Anopheles* and *Culex* mosquitoes [33–36].

In *Ae. aegypti* mosquitoes, L1014 has yet to be identified but several different *kdr* mutations have been detected in Southeast Asia; for examples, S989P, I1011M/V, V1016G/I and F1534C based on the mutation positions of house fly sodium channel [10, 37–41]. It is no surprise that more sodium channel mutations in resistant insects have been discovered taking the advantage of the convenience of molecular approach. Beside the identification of single point mutations, novel co-occurrence of mutations was also detected. As firstly reviewed by Brengues et al. [37], V1016G was closely related to S989P in Southeast Asian countries such as Indonesia, Myanmar, Thailand and Malaysia [9, 10, 39, 42], and this common phenomenon was believed to confer higher resistance. Co-occurrence of triple mutations V1016G, S989P and F1534C would result in even higher resistance to pyrethroids, as seen in *Ae. aegypti* populations from Myanmar [39]. The detection of these specific mutations, such as V1016G, S989P and F1534C, were affirmed to confer sodium channel resistance to pyrethroids and other associated mutations which are still yet to be inspected. It is very likely for the emergence of new *kdr* mutations when pyrethroids remained to be the primary insecticide-based interventions in the control of *Ae. aegypti*. Hence, these detections act as a crucial role in resistance management of *Ae. aegypti* by observing the occurrence of pyrethroid resistance.

It is remarkable that despite two distinct substitutions occur at the same amino acid position, V1016G and V1016I display different geographical variations with dissimilar response of *Ae. aegypti* sodium channel to pyrethroids. V1016G has been detected in Southeast Asia [37, 39, 40, 43, 44] while V1016I has been found in North and South America [45–47]. In recent times, the V1016I mutation was detected in Vietnam [41] and V1016I along with F1534C was detected in Ghana that there may be presence of migration event [48]. The detection of these point mutations in *Ae. aegypti* strains in continents they have never existed before requires further validation to confirm if the resistance is caused by migration or *de novo* (new) mutations. The evolution and distribution of pyrethroid resistance in *Ae. aegypti* populations is relevant when their eggs can endure desiccation for an extended period that permit them to put human travel to good use.

### Metabolic-mediated insecticide resistance

Metabolic resistance in *Ae. aegypti* involves alterations in a series of the expression of a complex group of enzymes, causing a rise in the detoxification process of insecticides. This resistance mechanism facilitates increased biodegradation of insecticides, with the help of enzymes such as GSTs, esterases and cytochrome P450 monooxygenases (P450 genes). A total of 160 P450 genes, 49 GSTs and 26 esterases have been revealed in an international genome project of *Ae. aegypti* [49]. Among these, cytochrome P450 genes are the enzyme family highly responsible for the development of pyrethroid resistance in *Ae. aegypti*. Therefore, cytochrome P450 genes are of interest to study since they are capable in metabolizing xenobiotics such as drugs, plant toxins and insecticides. One of the most noteworthy characteristics of P450 genes linked to enhanced metabolic detoxification of insecticides is the detection of a significant rise in P450 proteins and P450 activity due to the overexpression of insecticide-resistant insects P450 genes, which is closely associated with the development of insecticide resistance [50, 51]. However, identification of enzymes associated with insecticide resistance is difficult because of the presence of diverse group of P450 genes and high structural similarity in isoforms that makes identifying isoform related to resistance to be challenging. While molecular approaches in detecting *Vssc* gene mutations in pyrethroid-resistant *Ae. aegypti* are broadly documented, they are rare for P450 genes. *CYP9* family has been involved in pyrethroid resistance in the dengue vector *Ae. aegypti* [52]. *CYP9J22* and *CYP9M9* were the two P450 genes found with the highest transcript level of the resistant populations [52]. On a global scale, the most frequently found P450 genes are *CYP9M6*, *CYP6BB2*, *CYP9J26* and *CYP9J28*, which are commonly detected to be overexpressed in resistant strains in many studies, projecting their capability in metabolizing permethrin [25, 53, 54].

Esterase hydrolysis of pyrethroids resulting in the detoxification process has assumed to involve in metabolic resistance in some cases. This mechanism encompasses a series of reaction, such as gene amplification, upregulation, and/or coding sequence mutations. However, published data on which exact esterases in contributing to the presence of pyrethroid-resistant *Ae. aegypti* populations in Southeast Asia continues to be scarce. Elevated levels of GSTs are also commonly associated with insecticide resistance. Most of the GSTs are made of a super family structure of cytosolic-dimeric enzymes that are grouped into a total of six classes, namely Delta, Epsilon, Omega, Sigma, Theta and Zeta [55]. The two largest classes of GSTs for insects are Delta and Epsilon, provided that Epsilon class is often involved with resistance [56]. In *Ae. aegypti*, there are basically a cluster of eight sequentially arranged genes that mapped genetically to chromosome 2, supercontig 1.291 in the Epsilon GST class [56]. Recently, microarray studies have discovered some additional members of this gene cluster with elevated levels in *Ae. aegypti* populations resistant to insecticides [52]. However, their roles in metabolizing insecticides like pyrethroids are yet to be determined. Although esterases and GSTs are usually found overexpressed in pyrethroid-resistant *Ae. aegypti*, the causal of specific enzymes to the resistant populations from each of the enzyme family are still largely unverified up to now especially in Southeast Asia.

It is indisputably important to investigate metabolic-mediated insecticide resistance of *Ae. aegypti* to pyrethroids since co-existence of more than one mechanism is likely to occur, especially the largest endemic sites of DF Southeast Asia. In addition, efforts should be put in to the development of new insecticides, taking these enzymes related to insecticide resistance into consideration in the span of design procedure.

## Present situation of pyrethroid susceptibility in *Aedes aegypti* in Southeast Asia

Published literature with regards to the insecticide resistance profiles of *Ae. aegypti* against pyrethroids in most of the Southeast Asian countries are detailed as follows. Information were extracted from all publications documenting mortality, lethal concentration (LC_50_) and knockdown time (KT_50_) from bioassays of *Ae*. *aegypti* mosquito populations using merely pyrethroids. Since our main emphasis was chiefly placed on studies assaying pyrethroids against *Ae. aegypti*, other classes of insecticides tested and species other than *Ae. aegypti* found in the same publication were excluded and not reviewed in this study. *Kdr* assays used to observe the effect of control tactics on pyrethroid resistance were also studied. Distribution of different *kdr* mutations in *Ae. aegypti* in Southeast Asia are summarized in Table 1. Approaches employed to detect insecticide resistance when it first presents may allow timely associated management actions. Since there is a deficiency of studies on the metabolic resistance in some of the Southeast Asian countries, genes identified as overexpressed in resistant populations are reported only in countries with related publications. Publications from all years were retrieved, considered and incorporated into the review. Knowledge gaps which required attention are also identified in this review.

**Table 1:** Distribution of different *kdr* mutations in *Ae. aegypti* in Southeast Asia

Country	Mutation	Reference
Cambodia	F1534C	[60, 61]
	V1016G	[61]
Indonesia	V1016G	[10, 37, 44, 61, 63, 66]
	F1534C	[10, 44, 61, 63, 66]
	S989P	[10, 44, 63]
	V1016G/S989P	[10, 44, 63]
Laos	V1016G	[43]
	F1534C	[43]
Malaysia	V1016G	[9]
	F1534C	[9]
Myanmar	V1016G	[39, 61]
	S989P	[39]
	F1534C	[39, 61]
	V1016G/S989P	[39]
	V1016G/F1534C	[39]
	V1016G/F1534C/S989P	[39]
Philippines	na	
Singapore	F1534C	[25, 93]
	V1016G	[25, 40, 93]
Thailand	I1011V	[40]
	F1534C	[60, 61, 108]
	S989P/V1016G	[42, 112]
	V1016G	[37, 40, 61]
	V1016G/F1534C/S989P	[109]
Timor-Leste	na	
Vietnam	V1016I	[41]
	I1011V	[126]
	F1534C	[126]
	V1016G	[126]

*Abbreviation*: na, not available

### Cambodia

Although dengue is a major health concern in Cambodia [57], there are merely two known studies evaluating insecticide resistance status of *Ae*. *aegypti* in the country. The first related study was carried out using temephos (an organophosphate) at Phnom Penh and Kampong Cham in accordance with WHO standard bioassays [58]. Mosquito populations from Phnom Penh revealed temephos resistance whereas the latter populations were susceptible [58]. It is believed that temephos resistance in Phnom Penh populations led to another more comprehensive study encompassing Phnom Penh, Siem Reap, Kampong Cham and Battambang using both temephos and pyrethroids (permethrin and deltamethrin). Resistance to permethrin and deltamethrin were reported in *Ae. aegypti* populations from all the aforementioned study sites [59]. Therefore, a nationwide assessment is recommended to examine pyrethroid resistance in *Ae*. *aegypti* since Cambodia is a country bordering the dengue-endemic countries Thailand, Laos and Vietnam, so as to develop effective vector control methods to halt the spread of DF.

In Cambodia, the point mutations of the *kdr* gene related with pyrethroid resistance of *Ae. aegypti* were analyzed alongside with samples from other Southeast Asia countries in two international studies. The F1534C mutation was detected in the first study [60] whereas C1534 mutant alleles (with VV/CC patterns) were detected in the second study [61]. Further detection is highly sought after due to a deprived of nationwide studies that may possibly lead to the discovery of other unreported point mutations of the *kdr* gene. This helps to understand the nature of pyrethroid resistant in *Ae. aegypti* in this country, given that dengue is endemic year-round in Cambodia. Biochemical mechanisms of *Ae. aegypti* to pyrethroid resistance have not been reported.

### Indonesia

After a first occurrence in 1968, countless outbreaks of DF and dengue haemorrhagic fever (DHF) cases transmitted by the primary vector *Ae. aegypti* mosquitoes have dramatically increased in recent years. The latest statistic showed 71,668 DF human cases in 2015 [62], covering all 34 provinces of the country. In Indonesia, pyrethroids have been broadly used since the 1980s against *Ae. aegypti* but there has been a dearth of knowledge on published information. Earlier, some pioneer works reported that *Ae. aegypti* populations from West Java (Ciamis, Purwakarta and Bogor) and Bandung were resistant to permethrin, lambda-cyhalothrin, cypermethrin and d-allethrin [11, 37]. Despite high LT_90_ values being recorded [11], their resistant status regrettably should not be concluded. The standard susceptible laboratory strain used as a control in the study appeared to be resistant, led to an uncertainty of the resistant status. More recently, published data showed the presence of pyrethroid resistance in *Ae. aegypti* even after approximately 10 years of the pioneer studies [10, 44, 63]. Their susceptibility profiles suggested similar trends of resistance to pyrethroids [10, 44, 63], signifying comparable patterns of insecticide usage in these sampling sites.

Over the years, biochemical studies have acknowledged the role of detoxifying enzymes in the development of pyrethroid resistance in *Ae. aegypti*. The first small-scale test in determining the resistance mechanisms involved in pyrethroid-resistant *Ae. aegypti* was accomplished in 2007, comprising strains from Bandung, Palembang and Surabaya [12]. Adult *Ae. aegypti* from Bandung demonstrated the highest resistance level to both permethrin and deltamethrin, corresponding to the high levels of activity of enzymes tested, i.e. oxidases, esterase A and esterase B, that play roles in the development of resistance to pyrethroids. In contrast, another study [64] showed that larvae from most sampling sites in Cimahi were still susceptible to pyrethroids although Bandung situates in absolutely close proximity to Cimahi. Adult susceptibility profile may greatly correlate with larval susceptibility profile in some cases [7, 65]. However, it should be remembered that there is a difference between resistance score in larvae and adults because they may develop dissimilar resistant mechanisms through different mechanism pathways from larval to adult stages [14]. Thus, adult bioassay is still required to verify the resistance status.

Other than the responsibility of detoxifying enzymes in the insecticide resistance mechanisms, mutations in the *Vssc* gene should also be highlighted because they may act as markers for resistance monitoring. In Indonesia, *kdr* profiling of *Ae. aegypti* had recorded three point mutations related to pyrethroid resistance, namely the S989P, V1016G and F1534C mutations [10, 37, 44, 63, 66]. The discovery of the V1016G mutation was first reported by Brengues et al. [37] in the Semarang strain. Later, some findings were in agreement that all three mutations in *Vssc* gene were detected in *Ae. aegypti* populations from Yogyakarta, Central Java Province, Denpasar and Jakarta [10, 44, 63, 66]. The V1016G mutation was most frequently detected in high frequencies, remarkably associated with permethrin and deltamethrin resistance in *Ae. aegypti*. Previous reports discovered relatively low frequency of the F1534C mutation, which did not demonstrate significant contribution to the resistance development of pyrethroids in *Ae. aegypti* [10, 44, 63, 66]. As for the S989P point mutation, it generally co-occurred with the V1016G mutation, showing association on pyrethroid resistance development in *Ae. aegypti*. Furthermore, the frequency of the V1016G mutation occurred in *Ae. aegypti* in the Central Java has increased two-fold in just a decade, compared to a report generated earlier [37]. These preliminary detections of insecticide resistance will fundamentally aid in initiating appropriate control measures in delaying development of mosquito resistance, for instance rotation of insecticide or addition of catalyst to enhance the efficacy of insecticides in suppressing *Ae. aegypti* in Indonesia.

### Laos

Compared to the neighboring countries, there is a scarcity of study on pyrethroid resistance in *Ae. aegypti* in Laos. The first DF outbreak was recorded in 1998 in Khammouane Province [67]. Although *Ae. aegypti* has been stated to be the main vector of numerous mosquito-related diseases in Laos [67, 68], with 22,772 cases of dengue reported during 2010 [69], there is only a single report investigating pyrethroid resistance status of *Ae. aegypti* in the country thus far. Samples were collected from five provinces and populations involved in the study identified high level of resistance against permethrin, which contrast with deltamethrin that exhibited high susceptibility in *Ae. aegypti* [43]. Higher quantities of P450 genes were detected in field compared to reference strains but no particular *CYP* was mentioned. The frequencies of *kdr* mutations were low for the V1016G mutation (< 0.36) but relatively higher for the F1534C mutation (> 0.6 for majority of places). With a total of 18 provinces available in Laos and a reliance upon pyrethroids in *Ae. aegypti* control after the official ban of DDT in 2010 [70], more comprehensive studies are needed in Laos, as this knowledge is fundamental in managing vector control programmes.

### Malaysia

DF or DHF has been a critical public health concern in Malaysia at all times since the first outbreak in 1973. Regardless of extensive fogging operations with malathion in Malaysia as early as the 1970s, and followed by the replacement of pyrethroids like permethrin and deltamethrin in early 1998 until today [71], dengue remains number one infectious disease with a total of 101,357 cases reported in the country [72]. Numerous surveillance activities have been conducted to investigate insecticide susceptibility status of *Ae. aegypti*. The first study examining on the pyrethroid resistance level of *Ae. aegypti* in Malaysia was accomplished in 2001, using field strains from Kuala Lumpur, Selangor, Negeri Sembilan, Johor, Kelantan and Pahang. The urban strain of *Ae. aegypti* from Kuala Lumpur showed the highest resistance to permethrin, validating the high levels of esterase compared to the laboratory strain [73]. This result was in agreement with the findings performed after almost a decade. Permethrin resistance persisted in field strains of *Ae. aegypti* collected from dengue-endemic areas of Kuala Lumpur [7]. Both larval and adult bioassays of the field strains confirmed the development of tolerance towards permethrin with several folds higher than the laboratory strain [7], indicating high reliability on chemical insecticides to control *Ae. aegypti* in an unprecedented scale at the dengue hotspots. Piperonyl butoxide (PBO) was also reported to be effective in enhancing the effectiveness of permethrin through which strong correlations were confirmed between LC_50_ or LT_50_ values and oxidase levels for all strains [7], implying the involvement of oxidase activity in causing permethrin resistance in this mosquito species.

Another similar study performed at dengue-prone sites of Selangor documented resistance and incipient resistance of *Ae. aegypti* to permethrin (mortality ≤ 80%) and cyfluthrin (mortality 45–97.8%), respectively [8]. Although cyfluthrin was not used by the municipal in vector control programme, fluctuated resistance was detected and this may be attributed to the role of *kdr*. Screening of *kdr* is yet to be performed in Selangor but it has already accomplished and confirmed on the presence of *kdr* in Kuala Lumpur [9], which is very close to Selangor. DDT resistance was also detected over the course of the study [8], further supporting the role of *kdr* in both pyrethroid and DDT resistance due to the shared *Vssc* target site. GST activity exerted on DDT resistance has not been discovered in Malaysian mosquitoes including *Ae. aegypti* [71, 74]. The widespread of insecticide resistance pays full responsibility to failures in vector control programme. *Aedes aegypti* populations from mainland Penang were also highly resistant to lambda-cyhalothrin [75]. These outcomes were supported by the fact that pyrethroids have been broadly sprayed in the study areas for more than 10 years. Of several studies conducted in the country, insecticide susceptibility status of permethrin was often tested against *Ae. aegypti* in Malaysia because this insecticide is one of the main adulticides used in Malaysia [76]. Moreover, the development of permethrin resistance at a higher rate than malathion and temephos [77] might be closely associated with gene activation due to the exposure to insecticidal pressure.

More recently, despite discrepancies of insecticide susceptible status found in the field strains of *Ae. aegypti* from Kuala Lumpur, Penang, Johor Bharu and Kota Bharu tested against permethrin and deltamethrin, the mosquito strains from Kuala Lumpur remained to exhibit the highest resistance levels [9]. Synergist assays with PBO showed that metabolic resistance mechanisms played key role in certain strains. This particular study is the first report in Malaysia in characterizing the *kdr* resistance in Malaysian populations of *Ae. aegypti*. The V1016G and the F1534C mutations were detected, with the F1534C mutation closely associated with pyrethroid resistance whereas the V1016G mutation co-occurred to contribute to the additive effect of pyrethroid resistance in Malaysia [9]. A microarray-based genome-wide transcriptional analysis discovered that metabolic resistance of pyrethroid resistance in *Ae. aegypti* populations is predominantly caused by overexpressed of the cytochrome P450 genes (*CYP9J27*, *CYP6CB1*, *CYP9J26* and *CYP9M4*) [32]. However, more characterization work on the cytochrome P450 family must be carried out to understand the precise roles of these genes in contributing pyrethroid resistance in *Ae. aegypti*.

As far as we know, Malaysia is one of the two countries in Southeast Asia tested on the efficacies of the commercial mosquito coils against *Ae. aegypti*. The mosquito coil is one of the most extensively-used anti-mosquito household spatial repellents in Malaysia, accounting for the low costs and easy availability on-shelf countrywide [78–81]. In 1996, the first study assayed on the efficacies of mosquito coils containing active ingredients like d-allethrin (0.2% w/w) and d-trans allethrin (0.1% w/w) against laboratory *Ae. aegypti* were accomplished, revealing adequate knockdown effects but with minimal mortalities for both coils [78]. After almost a decade, a similar study was conducted with the use of d-allethrin (0.2% and 0.3% w/w) and d-trans allethrin (0.1% and 0.2% w/w) on laboratory strain of *Ae. aegypti* [82]. Dosage increment of the active ingredients in mosquito coils, which are 0.3% w/w of d-allethrin and 0.2% w/w of d-trans allethrin were proven to show higher repellent efficacies and mortality [82]. However, this result requires further verification on field strains in natural settings. In the past decades, efficacy studies were performed on the laboratory strains of *Ae. aegypti*. Until recently, insecticidal activities of Malaysian commercial coils have been assessed on field strains of *Ae. aegypti* in a nationwide-scale. *Aedes aegypti* from a total of 11 states were discovered to be resistant to mosquito coils containing active ingredients of d-allethrin, d-trans allethrin, metofluthrin and prallethrin, according to the resistance index recommended by the WHO [83]. Since there is a wide array of insecticides used intensively in the country to curtail disease transmission, either operations conducted by the Ministry of Health, private companies or even at household level [84], it is utmost important to assess regularly on the efficacies of insecticides used to guarantee effective vector control programme.

### Myanmar

The first documented outbreak of DHF was in 1969 and then the outbreaks gradually increased to 12 out of 14 states in Myanmar [85]. This disease was documented with a high of 83,381 cases, causing 3243 deaths between 1970–1991 [85]. No sign of decrement in DHF incidence with 5621 cases in 2005 to an approximate two-fold of 11,049 cases in 2006 [86], despite persistent control efforts made by the Myanmar government since 1968 [85]. Although the significance of the disease requires immediate attention, there is only a single report on the ecological study of *Ae. aegypti* focusing on the southern part of Myanmar [86]. There is still an unavailability on the bioassay studies of the status of pyrethroid resistance in *Ae. aegypti* in the country.

There is an absence of study to date, regarding the biochemical mechanisms of *Ae. aegypti* to pyrethroid resistance. In 2014, a project was initiated to evaluate the types and frequencies of point mutations of the *kdr* gene related with pyrethroid resistance of *Ae. aegypti*. It was revealed that V1016G and S989P mutations were extensively scattered in Yangon city with high frequencies of 84.4% and 78.8%, respectively [39]. Another widely scattered point mutation identified with relatively low allelic frequency of 21.2% was F1534C [39]. Other than three single point mutations were detected in Yangon city, three patterns of co-occurrence of point mutations were also identified with widely distributed homozygous V1016G/S989P mutations (65.7%), as well as a small number of homozygous V1016G/F1534C mutations (2.9%) and homozygous V1016G/F1534C/S989P (0.98%) [39]. It should be noted that the observed types and frequencies of resistance alleles may be very well representing the Yangon city, as samples were collected from seven townships. However, it may not be a decent approximation of types and genotype frequencies of the country because the size of Yangon city (598.75 km^2^) is relatively small compared to Myanmar (676,578 km^2^). Therefore, sampling is needed throughout the country to ensure the surveillance of resistance genes in wild *Ae. aegypti* populations are comprehended.

### Philippines

There appears to be an agreement in literature that *Ae. aegypti* was introduced into the Philippines approaching the end of 19th century and followed by the proliferation of this dengue vector [87]. However, there are only two known studies evaluating the insecticide resistance of *Ae. aegypti* to pyrethroids in the Philippines. In the first study, *Ae. aegypti* from Luzon Island, Manila was confirmed to be susceptible to dieldrin even after eight years of insecticide application in the country since 1959 [88]. Currently, the latest susceptibility test of *Ae. aegypti* with the use of pyrethroid was completed in 1997 in Cebu city. It was proven that treatment of curtains with permethrin was an effective vector control measure [89]. These preliminary studies facilitated in verifying the development of resistant strains in the country but the information may not be up to date, and related studies are recommended in the future because DF is endemic in neighboring countries like Malaysia. Susceptibility tests of different pyrethroids and related resistance mechanisms should be attempted on different field populations of *Ae. aegypti* in the country to confirm insecticide susceptibility status and guarantee the representativeness of the information generated since those insecticides have been put to use for several decades.

### Singapore

It is clear that *Ae. aegypti* mosquitoes are the primary vectors of DF in Singapore, with the first dengue case reports in the 1960s [90]. In the 1970s, pyrethroids were first introduced to the country to deprive the populations but permethrin resistance was identified in field *Ae. aegypti* later [91]. Subsequently, an assessment in 1999 revealed a 12.9-fold of RR_50_ of field *Ae. aegypti* against permethrin [13]. Resistance of *Ae. aegypti* to cypermethrin was also reported in a study between 2004–2007 [92]. More recently, the detection of resistance of *Ae. aegypti* to pyrethroids were further verified by two comprehensive nationwide insecticide resistance studies on larvae tested with permethrin and etofenprox, as well as adults with cypermethrin, deltamethrin and etofenprox [14, 15]. Despite the a few decades of gap between these studies, high resistance to pyrethroids remained to persist in the Singaporean *Ae. aegypti* populations and they continued to thrive, portraying the widespread or probably inappropriate use of pyrethroids ever since they were first introduced in Singapore. The use of synergists (piperonyl butoxide, *S*,*S*,*S*,-tributyl phosphorotrithioate, and triphenyl phosphate) was also deemed to be insignificant to the insecticides used in these studies to increase toxicity, signifying negligible roles of metabolic-based resistance when further biochemical investigation discovered that detoxifying enzymes such as monooxygenases, esterases, and GSTs failed to contribute to pyrethroid resistance [14, 15]. The ineffectiveness of synergists proposes that molecular work is recommended to further investigate the mechanisms contribute to the high pyrethroid resistance exhibited in local *Ae. aegypti*.

Other than fogging, space spraying, chemical treatment and source reduction as dengue vector control methods, deltamethrin-treated net is an alternative. However, its efficacy was proven to be unsatisfactory against field *Ae. aegypti* in a recent study [93]. Three molecular investigations have evaluated the roles of individual and multiple *Vssc* gene mutations in contributing to the developed pyrethroid resistance in Singaporean *Ae. aegypti*: F1534C, S989P+V1016G and F1534C+V1016G [25, 93, 94]. However, in-depth study showed that G1016 alleles independently pay more responsibility to *Vssc* gene insensitivity than C1534 alleles independently [25, 94]. To the best of our knowledge, S989P+V1016G+F1534C triple mutations in *Vssc* gene exhibited in the local *Ae. aegypti* were first reported in Singapore. They exerted the largest pressure in channel sensitivity to permethrin and deltamathrin by 1100-fold and 90-fold [94], implying the likelihood of reduced efficacy to pyrethroid insecticides. There is also a need of an immediate attention to monitor the occurrence of triple mutations in *Vssc* gene of field *Ae. aegypti* populations worldwide, so that the challenge of mosquito vector control will not further exacerbated. The significance of P450-mediated resistance is mirrored by the highly-resistant *Ae. aegypti* Singapore (SP) strain, showing 1,650-fold of resistant to permethrin and PBO has reduced the resistance by 48-fold [25]. P450 genes (*CYP4C50*, *6BB2*, *6F2*, *6F3*, *6Z7*, *6Z8*, *9M4*, *9M5* and *9M6*) were also discovered to be overexpressed in adult females and males [25]. Precise and reliable molecular diagnosis approach to identify metabolic enzymes conferring pyrethroid resistance in *Ae. aegypti* populations should be emphasized to elucidate the contribution degree of P450 genes to field-resistant strains from distinct areas.

### Thailand

Thailand is the country with the most studies evaluating on the insecticide resistance of pyrethroids to *Aedes aegypti* in Southeast Asia, reflecting a substantial geographical bias as this species is widespread in many countries in this sub-region. Thailand first experienced outbreaks of dengue hemorrhagic fever in 1958 and the disease has since then distributed nationwide [95]. To date, DF or DHF remain a severe health threat in Thailand regardless of unrelenting vigilance in vector control programmes. Reliance has been on the carbamate, organochlorine and organophosphate insecticides since 1950 [17] and the use of synthetic pyrethroids then dominated the market since 1992. Pyrethroid-based formulations with 12 distinct active ingredients are commercially available to all household levels countrywide to control mosquitoes in response to their low price, quick knockdown effect and are relatively safe for human contact because of low mammalian toxicity [17]. Years of routine contact with these insecticides have induced high levels of resistance in *Ae. aegypti*.

To understand the insecticide susceptibility profile of Thai *Ae. aegypti* to pyrethroids, there have been several published pioneering works available, but specific geographical restrictions were observed whereby they were conducted in small confined areas during previous decades. Increasing tolerance or resistance to different types of pyrethroids, namely deltamethrin, permethrin, dieldrin, bioallethrin, bioresmethrin or alphacypermethrin, has been reported in larval and adult *Ae. aegypti* in Thailand [16–18, 96–101]. The absence of large scale evidence-based understanding of the knowledge between the susceptibility profile and insecticides used may largely hinder the effect of dengue control efforts. A comprehensive study evaluating the insecticide susceptibility level will aid in better efficiency in programme-planning to target the disease vector. More recently published data on pyrethroid resistance in Thai *Ae. aegypti* addressed the issue that all 32 strains of *Ae. aegypti* were discovered to be resistant to permethrin, ranging between 4.0–56.4% [102]. The frequency of susceptibility to deltamethrin in this species showed more than 98% of mortality to deltamethrin in the majority of the populations, with incipient resistance detected in minor populations. Conversely, all 32 strains of *Ae. aegypti* were entirely susceptible to lambda-cyhalothrin with 100% mortality. Significantly high levels of permethrin resistance were documented by [18] (5% mortality) and [96] (2–9% mortality) which contrast with this particular study. This may attribute to the differences in sampling sites and the alterations in levels of exposure to permethrin.

Personal protection tools, for instance mosquito coils, are extensively used in Thailand but these products have never been thoroughly studied. Although literature presents on the efficacies of commercially available mosquito coils, up to now, information about detailed assessment on these coils to field populations of *Ae. aegypti* remains to be sparse. These studies are irrefutably important when mosquito coils are the top choice of household insecticide formulations worldwide other than aerosols, vaporizing mats and liquid vaporizers. The first report was completed in 2008 in Thailand, showing a phenomenon of tolerance of *Ae. aegypti* to dl, d-T80-allethrin, d, d-T-prallethrin and methoxymethyl-tetrafluorobenzyl tetramethyl-cyclopropanecarboxylate (K-3050) under semi-field condition [103]. A similar study was conducted with a total of four field strains of *Ae. aegypti* tested against the same active ingredients, employing 25 m^3^ semi-field test system, topical application test and field efficacy test [104]. Most of the strains were recorded to be tolerant to the mosquito coils tested but pyrethroid resistance may have already developed since noticeably large gaps were observed between the timeframe of sample collection and the course of the study, with up to 31 years gap. It is recommended that, when possible, mosquito test populations should be acquired with shorter gap to prevent bias in the outcomes. The latest related study was accomplished in 2009 using 25 m^3^ semi-field test and topical application test, demonstrating extremely low susceptibility to dl, d-T80-allethrin in some field strains [105] and that they may have already developed resistance now. All three studies yielded similar outcomes with low allethrin susceptibility, but it should be noted that direct comparison of results should be avoided due to different testing approaches used. Standardization of method is needed to avoid significant data deviation from a study to another. The knockdown time may vary in regard to types of method employed, that according to Chadwick [106], results carried out in cylinders, small chambers and large rooms are distinct. Moreover, associated assessments on the efficacy of mosquito coil to Thai *Ae. aegypti* are also needed.

A simple colorimetric assay based on Heated Oligonucleotide Ligation Assay (HOLA) *kdr* assay from past studies was developed to detect substitutions within domain II, subunit 6. i.e. Met1011, Val1011, Ile1016, and Gly1016 [40]. The V1016G mutation was evidently detected in high allele frequency of 0.23 throughout Thailand in pyrethroid-resistant *Ae. aegypti*, and that the I1011V mutation to be the minority with an allele frequency of 0.14 [40]. Despite only a thermal cycler being involved in this assay, extra reagents are needed which contributes to the increment of cost. An allele-specific polymerase chain reaction (AS-PCR) assay was then developed to detect the V1016G mutation which was shown to be reliable [107]. Homozygous 1016G mosquitoes were found to be common in Thailand and showed higher survival rates than either heterozygous or wild-type (1016V) mosquitoes upon deltamethrin exposure, indicating this particular mutation confidently related to deltamethrin resistance. Subsequently, the F1534C mutation was discovered as a novel amino acid mutation in 2010 in permethrin-resistant *Ae. aegypti*, and was reported to play a significant role in contributing permethrin resistance in multiple field strains [108]. The wide distribution of this mutation led to the development of high-throughput molecular tools, namely TaqMan single nucleotide polymorphism (SNP) genotyping and an AS-PCR assay, which proved to be consistent in detecting the F1534C resistance mutation in the permethrin-resistance *Ae. aegypti* populations [108]. Recently, a multiplex polymerase chain reaction (PCR) was developed to detect both V1016G and F1534C *kdr* mutations in *Ae. aegypti* through a single-reaction protocol [61]. This method was evidenced to depict high sensitivity and specificity in detecting the aforesaid *kdr* mutations, enabling the monitoring of the frequency of mutant alleles across dengue-endemic countries at ease with reduced time and cost.

Other than the discovery of singly-occurred mutations, the novel co-occurrence of the S989P mutation and the V1016G mutation were detected in Thai deltamethrin-resistant *Ae. aegypti* [42]. The 1016G mutation usually coexists with 989P mutation but there is also an absence of 989P mutation in some Thai populations which were homozygous for 1016G [37]. Since some studies showed that the V1016G mutation has been reported in the absence of the S989P mutation, it is likely to hypothesize that this point mutation acts as an additive mutation [42]. Furthermore, the V1016G/F1534C/S989P mutations have been confirmed in Thailand, demonstrating an additive effect on deltamethrin sensitivity which gives high level of resistance [109]. Recent work has also emphasized that the variations of point mutations exerted on pyrethroid-resistant *Ae. aegypti* revealed different response to insecticides. Thermal fogging spray with deltamethrin and PBO synergist killed all resistant genotypic mosquitoes in indoors [110]. In contrast, the outdoor spray displayed minor impact on the G1016 homozygous mosquitoes, partially killed the G1016/C1534 double heterozygous mosquitoes and triggered high mortality in the C1534 homozygous mosquitoes [110]. Further associated studies should be conducted to understand other polymorphisms complementing the *Vssc* mutation and how they are related to the resistance phenotype when selection pressure is extrapolated to act on the survival of pyrethroid-resistant *Ae. aegypti*. Moreover, further verification is also needed to make implications that the aforesaid mutations are linked to deltamethrin resistance. Other than the target site insensitivity mechanism acts upon the pyrethroid resistance in *Ae. aegypti*, a previous study addressed the absence of the 1016G mutation in deltamethrin resistance mosquito strains and revealed the role of mixed function oxidases in conferring the resistance [16]. Thus, the contributions of metabolic mechanisms in these wild-type individuals upon deltamethrin exposure earn further attentions. This overlaps the study of Somwang et al. [111], which described oxidative enzyme systems also involve in pyrethroid resistance in *Ae. aegypti* in Thailand. Another study showed that biochemical assays are very much required when a difference of low *kdr* resistant allele frequency of S989P and V1016G with high level of permethrin resistance was reported in field strains of *Ae. aegypti*, which this implies detoxification enzymes show the possibility to be involved in insecticide resistance mechanisms [112].

Biochemical assays used to detect enzymes metabolizing insecticides have been available for over three decades to monitor insecticide resistance of *Ae. aegypti* in various countries. In Thailand, the first related study was accomplished in 2002 using laboratory bred artificially selected resistant *Ae. aegypti*. GST and esterase activities were detected to be only marginally higher relative to the susceptible strain, signifying that both enzyme groups show no major role in permethrin resistance [113]. In contrast, the subsequent biochemical assay of metabolic enzymes tested with field *Ae. aegypti* to pyrethroid resistance revealed esterase, monooxygenase and GST are closely associated with permethrin resistance whereas deltamethrin resistance is related to esterase and monooxygenase [21]. Later, a number of studies proposed that an elevated level of mixed function oxidase was usually in association with pyrethroid resistance [16, 114]. Moreover, considerable escalated levels of esterases and GSTs were stated in some field strains of pyrethroid-resistant *Ae. aegypti* [114]. The latest study demonstrated that the aforementioned enzymes were either unrelated or merely contributed partially in pyrethroid resistance [115]. In regards to the discrepancies of these enzymes as contributory factors to pyrethroid resistance in *Ae. aegypti*, it should be noted that *in vitro* experiments may not reflect the *in vivo* circumstances of insect metabolizing insecticide molecules and thus, field studies including numerous variables should be considered in the future.

### Timor-Leste

Timor-Leste is not excluded from vector-borne diseases transmitted by *Aedes* mosquitoes, due to its generally warm and humid climate which is conducive for their growth. Dengue cases were first reported in 2003, followed by an outbreak resulted in 933 cases and 37 deaths in 2005 in Dili, Bobonaro and Baucau [116, 117]. Although *Ae. aegypti* was extensively spread in the country [116], there was only single international project carried out, by Frances et al. [118] who employed bottle bioassay to examine insecticide resistance in *Ae. aegypti* and the respective biochemical mechanisms using pyrethroids (permethrin, resmethrin, lambda-cyhalothrin). These insecticides were in line with the other insecticides used more than a decade ago for vector control programmes in the country as stated by Whelan & Petitt [117]. Since pyrethroid resistance was detected in *Ae. aegypti* collected from a single site in Dili with elevated levels of esterases, there is a need in investigating the efficacies and mechanisms contributed to the resistance of pyrethroids towards the *Ae. aegypti* populations in Timor-Leste.

### Vietnam

DHF was first documented in Hanoi, Vietnam in 1958 [119], followed by the first and second outbreaks recorded in 1960 and 1963, respectively, in South Vietnam [120]. Vietnam, like all the other Southeast Asian countries, utilizes chemical insecticides as the primary tool in ceasing spread of the vector of DHF, *Ae. aegypti*. Although *Ae. aegypti* was introduced to the country in 1915 [121] and DHF has been reported to be endemic in Vietnam for decades [122], there are insufficient amount of studies available in evaluating the efficacies of pyrethroids to *Ae. aegypti* and their related mechanisms [41, 122–126].

The first insecticide resistance study of *Ae. aegypti* was conducted in 1998 and 1999, showing that the mosquitoes were resistant to few pyrethroid insecticides in several locations of Central Highlands and Nam Bo [122]. Later, a similar but more comprehensive study was completed in 2004, encompassing four regions of Vietnam, and revealed that *Ae. aegypti* from numerous locations in the Centre and North regions were susceptible to pyrethroids but those from South and Central Highlands were more resistant [124]. This was believed to be related to the frequent and prolonged use of pyrethroids in the highlands for the *Ae. aegypti* control programme [124]. Apart from the aforementioned factor that may influence the susceptibility status of *Ae. aegypti* to pyrethroids, mosquitoes from places with high populations develop higher resistance than those from outskirts; Huber et al. [123] showed that *Ae. aegypti* in cities with high populations, like Ho Chi Minh City, developed higher resistance than those from the outskirts or Long An Province. Other than inspecting previously reported dengue hotspots, these pioneer studies provided insights into pyrethroid resistance in *Ae. aegypti* and reported that *Ae. aegypti* populations in the country had already developed resistance to some pyrethroids.

The most extensive nationwide assessment of pyrethroid resistance in *Ae. aegypti* was completed in 2009, approximately a century after this disease vector made its way to Vietnam. A total of 527 collection points were involved from northern to southern Vietnam [125]. A simple bioassay of fourth instar larvae using glass vials was developed to detect knockdown susceptibility [125]. The most notable outcome of this study demonstrated a pronounced increment of resistance in *Ae. aegypti* to d-allethrin with decrease in the latitude of sampling sites [125], corresponding to the findings of an earlier study [124]. Analysis of point mutations was further investigated and the F1534C mutation was verified to be the chief point mutation that gave rise to high resistance in the specimens collected from the South, whereas the heterozygous V1016G mutation was very low in frequency [126]. Although low frequencies were detected in the North and the percentage of homozygous F1534C remained low (7.4%), unrestrained use of photo-stable pyrethroids that persists in the environment may induce selection pressure for this point mutation, which will result in more resistance offspring. With 21,000 liters of pyrethroids used in dengue control operations in 20 southern provinces in 2007 [127], it is no surprise that *Ae. aegypti* populations from Vietnam have developed insecticide resistance. Therefore, the use of PBO as a synergist was considered to increase the efficacy of detalmethrin to *Ae. aegypti* from Nha Trang [41]. The study subsequently proved that synergists might play crucial role in *Ae. aegypti* control programme [41]. Moreover, Nha Trang strain housed multiple resistance, the overexpressed P450 gene *CYP9J32* relative to the susceptible strain was found capable in metabolizing deltamethrin effectively, as well as two homozygous *kdr* mutations which were the I1011V (100%) mutation and the V1016I (67%) mutation. Attention should be paid to the detection of the V1016I mutation in Vietnam when this point mutation was only circumscribed in the continent of America in the past [38, 45, 128]. Thus, consistent monitoring of insecticide resistance is required.

## Future challenges and perspectives

When development of resistance has become a worldwide issue, there are alternatives to control *Ae. aegypti*. Environmental control in Southeast Asia involves source reduction and the use of mosquito traps or screen net covers [129–132]. Standing water and unnecessary containers, in both indoor and outdoor conditions, should be eliminated to prevent breeding of mosquitoes, specifically *Ae. aegypti* and other container breeders [79]. Containers that performed functions in daily life should be screened or properly covered. Source reduction should also include getting rid of natural habitats that collect water, such as bamboo stumps and tree holes to avoid the breeding of *Ae. aegypti*.

To diversify the choices of vector control, research has been reinforced in developing pathogenic organisms to combat dengue vector. In biological control, natural enemies are either predators, microbes or parasites [79]. Examples of biological control in Southeast Asia include the participation of predaceous aquatic insects as natural enemies to suppress mosquito populations by predating on mosquito larvae as food source [57, 133, 134] or the use of the entomopathogenic bacteria *Bacillus thuringiensis* var. *israelensis* to destroy the gut lining of mosquito larvae [130, 135]. Hypothetically, predaceous animal species should result in reduction of mosquito populations, but there is limited evidence regarding tangible proof of related declining disease burdens. Therefore, it should be noted that these methods would work well alongside chemical insecticides but should never be employed as a sole control tactic during epidemic outbreaks of dengue when immediate elimination of disease vectors should be prioritized.

Another biological control method involves the release of *Ae. aegypti* infected with *Wolbachia*. These naturally-occurring bacteria infect a wide range of arthropods and nematodes but are absent in *Ae. aegypti* [136]. To decrease dengue transmission, *Wolbachia* from naturally infected organisms are vertically transmitted into *Ae. aegypti*. This causes sterility *via* cytoplasmic incompatibility, resulting in eggs without progeny when an uninfected female *Ae. aegypti* mates with a *Wolbachia-*infected male [137]. *Wolbachia* has drawn much attention and field trials are ongoing in several countries such as Australia [138], Vietnam [139] and Indonesia [140]. Despite wMel *Wolbachia* showing positive result in lowering the incidence of dengue in human populations in northern Australia [138], wMelPop *Wolbachia* with even more significant resistance to DENV infection failed to establish successfully in Australia and Vietnam. Hence, more progressive studies pertaining to the large-scale field release of *Wolbachia*-infected *Ae. aegypti* are essential before this approach would show success in dengue control programmes throughout dengue-endemic countries.

It is irrefutable that chemical insecticides remain the primary vector-control intervention, specifically during an emergency epidemic. When *Ae. aegypti* exhibited a propensity to develop resistance to countless groups of chemical insecticides leading to loss of functions, insect growth regulators (IGRs) may likely reduce resistance developing. The discovery of IGRs is in accordance with the knowledge of their growth, development, function and behavior. Thus, the use of IGRs has the likelihood to conquer the market of many other insecticides, and Lau et al. [141] reported that this group of insecticides poses encouraging results to control the field populations of *Ae. aegypti*, specifically cyromazine.

Pyrethroid resistance in Southeast Asian *Ae. aegypti* should not be overlooked. Many countries rely on the interventions of chemical insecticides to control the mosquito vectors of dengue. In this review of literature, several issues that highlight the need for immediate attention are addressed. Undeniably, these studies demonstrated a strong tendency of lopsided geographical distribution in published reports with more than half published from Thailand, Indonesia and Malaysia. Although studies retrieved from the database reflected the burden of DF, it is believed that some dengue-endemic countries such as Vietnam and Myanmar may have restricted the accessibility of the database without releasing to the public. This leads to difficulty in deciding on suitable insecticides for dengue control and, therefore, a platform with insecticide resistance status of different *Ae. aegypti* populations in a homogenous format would critically solve related challenges and ease future vector control planning. In certain cases, uniformity of protocols was neglected. Examining the broad range in the methodologies utilized to record and analyze resistance or susceptibility data of various studies is very challenging. Thus, only some attempts were made to specify the level of susceptibility that the standardization of methods in all of the studies would contribute to reliable comparison of the outcomes. Method of recording insecticide resistance should be consistent, taking LC_50_/LT_50_/KT_50_ or other similar set of data for instance, though actual values may still have to be provided for comparison in some circumstances.

In addition, the use of the revised version of World Health Organization (WHO) guidelines was not practiced in all the studies. WHO has initially published two different databases on the diagnostic dosages of distinct insecticides [142, 143]; there are still some studies that employed the original diagnostic doses to examine the susceptibility status of *Ae. aegypti* [8, 13, 18], while some have already opted for the newly revised guideline [97, 99]. In recent times, WHO has released the latest version of test procedures to detect insecticide resistance for both *Aedes* larvae and adults [144]. All researchers are recommended to adhere to the changes made by WHO on the guidelines to evaluate the resistance status of field *Aedes* population. Furthermore, future studies should pinpoint on widening the number of sampling sites on a wide array of active ingredients of the pyrethroid group that are yet to be tested in most cases, including resmethrin, bifenthrin, flumethrin and tralomethrin. These may have potential in effective control of *Ae. aegypti* or cross-resistance within the active ingredients of the pyrethroid group that may have already occurred whereby the last resort would be abandoning of the pyrethroid group of insecticides. The prevalent use of pyrethroid-based mosquito coils calls for further validation on their efficacies across Southeast Asia. As of now, little has been reported on the efficacies of these mosquito coils to *Ae. aegypti* populations.

Efforts of understanding the aforementioned mechanisms on *Ae. aegypti* are urgently required, especially in some dengue-endemic countries with unavailability of associated reports, as vector control against *Ae. aegypti* continues to be the corner stone in preventing dengue transmission and outbreak control. Bioassays are only capable in detecting resistance that has already existed in a strain, while diagnostic assays can detect resistance once it appears that this may avoid the failure in vector management. *Kdr* assays have long been used for detecting point mutations to observe the effect of control tactics on insecticide resistance, but it is crucial to note that these molecular assays of target site resistance should also not replace bioassays until other resistance mechanisms, e.g. metabolic resistance can be complemented by comparable assays. A major advantage of the molecular approach is that sequencing of *Ae. aegypti* genome aids in progressive study on insecticide resistance mechanisms and further research should focus on searching for new diagnostic markers of pyrethroid resistance in dengue vectors. Biochemical assays in detecting enzymes linked to pyrethroid resistance have been utilized in several Southeast Asian countries, because these assays deliver important information for estimating resistance. However, the short of specificity and sensitivity of some assays may lead to complications in analyzing data. As for the addition of synergists, such as PBO used to examine the significant of metabolic resistance, the protocol can be very problematic as a huge amount of alive *Ae. aegypti* populations will be needed. Hence, the above-mentioned challenge in biochemical assays are more difficult when analyzing the use of synergist data.

## Conclusions

Across Southeast Asia, dengue appears to be endemic. Thus far, the unavailability of data regarding pyrethroid resistance in *Ae. aegypti* in some parts of Southeast Asia, such as Brunei Darussalam, Christmas Island of Australia and The Andaman/Nicobar Islands of India, necessitates immediate research to be conducted. As for the reported countries, resistance status of many *Ae. aegypti* populations to pyrethroids demonstrates the need for more effective control strategies, possibly the intervention of new insecticides. The fact that either migration or *de novo* mutations is creating issue regarding the spread of *kdr* mutations that were once occurred locally are now discovered in different continents, despite geographical separations, should be highlighted. More efforts are bound to combat insecticide resistance to stop the spread of diseases and vectors. However, it is understandable that this could be a difficult undertaking, because a substantial amount of funds is needed. Therefore, vector control success will very much count on policymakers, academics and scientists.

## Abbreviations

AS-PCR: Allele-specific polymerase chain reaction
DENV: Dengue virus
DF: Dengue fever
DHF: Dengue hemorrhagic fever
GST: Glutathione S-transferases
IGR: Insect growth regulator
*kdr*: Knockdown resistance
PBO: Piperonyl butoxide
PCR: Polymerase chain reaction
SNP: Single nucleotide polymorphism
*Vssc*: Voltage-sensitive sodium channel
WHO: World Health Organization
